# Supplementary material for: TREM1 rs2234237 (Thr25Ser) Polymorphism in Patients with Cutaneous Leishmaniasis Caused by Leishmania guyanensis: A Case-Control Study in the State of Amazonas, Brazil
Source: Pathogens. 2021 Apr 20;10(4):498. doi: 10.3390/pathogens10040498 (PMC8074324; doi:10.3390/pathogens10040498)
Supplement: Supplementary file 1 [file pathogens-10-00498-s001.zip › pathogens-1101723 supp fig S1.pdf]

Supporting information

S1A Fig. *TREM1* rs2234237 A/T (Thr25Ser) influence on circulating cytokines plasma levels.

S1B Fig. *TREM1* rs2234237 A/T (Thr25Ser) influence on circulating cytokines plasma levels.

S1 Table. Circulating plasma cytokines levels in patients with CL and healthy controls adjusted by sex and age.

S1A Figure

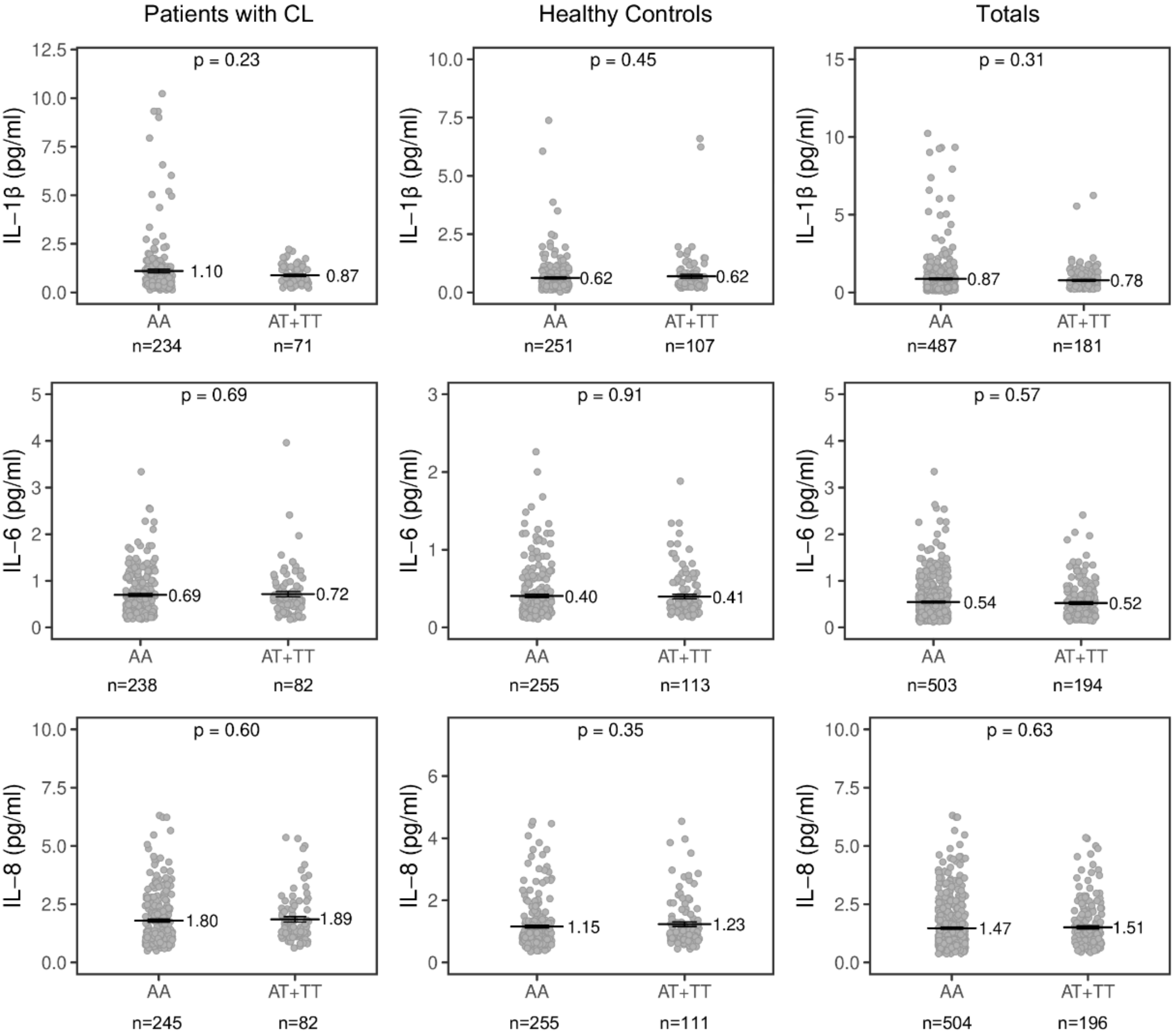

S1A-B Figure *TREM1* rs2234237 A/T (Thr25Ser) influence on circulating plasma levels of IL-1β, IL-6, IL-8, IL-10, MCP-1 and TNF-α in patients with CL, healthy controls and total subjects adjusted by sex and age. The black bars represent the mean of concentrations in picogram per milliter (pg/ml), whereas error bars represent standard error of mean. p-value <0.05 is considered significant.

S1B Figure

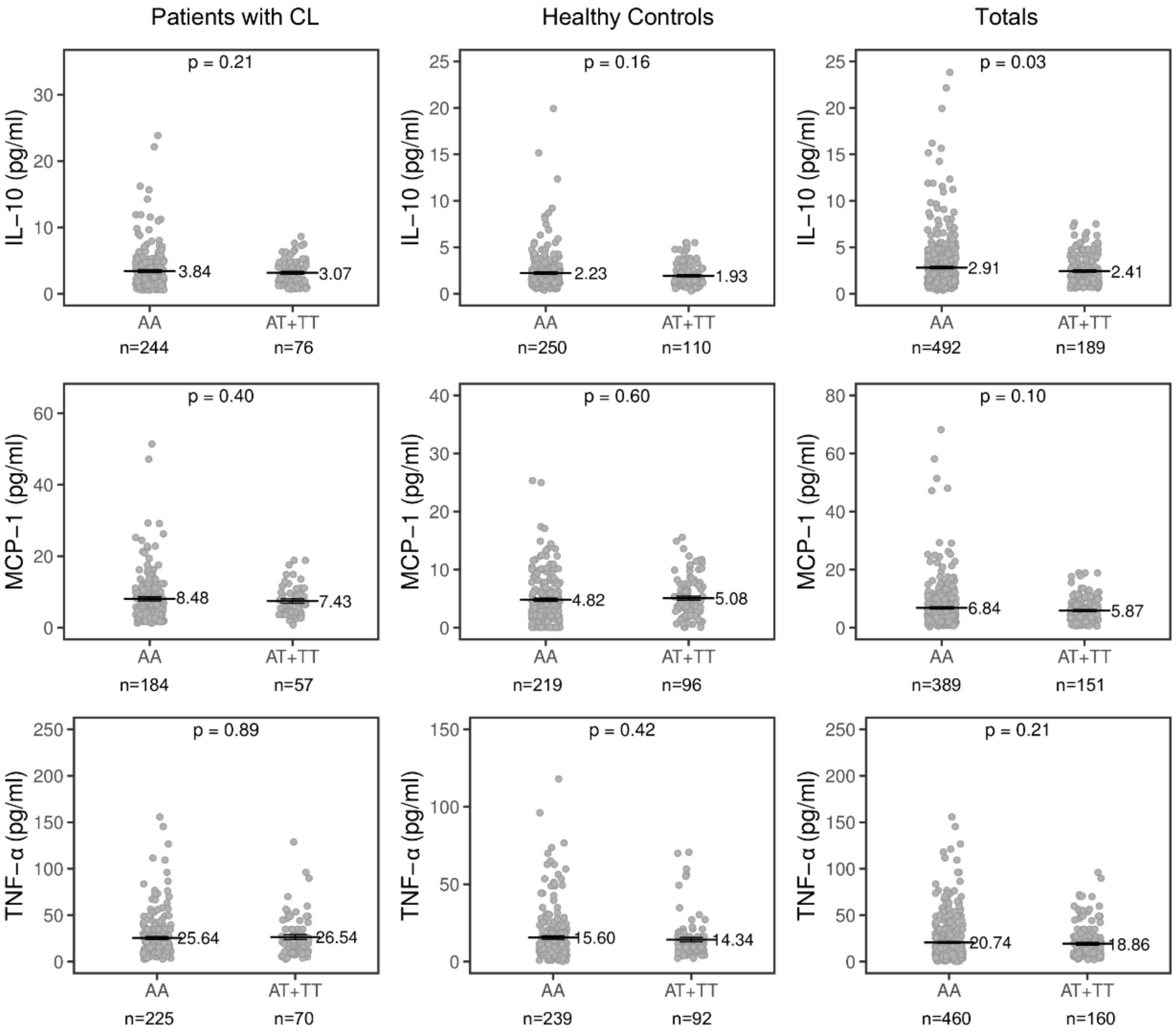

S1A-B Figure *TREM1* rs2234237 A/T (Thr25Ser) influence on circulating plasma levels of IL-1β, IL-6, IL-8, IL-10, MCP-1 and TNF-α in patients with CL, healthy controls and total subjects adjusted by sex and age. The black bars represent the mean of concentrations in picogram per milliter (pg/ml), whereas error bars represent standard error of mean. *p*-value <0.05 is considered significant.
