# Supplementary material for: TREM1 rs2234237 (Thr25Ser) Polymorphism in Patients with Cutaneous Leishmaniasis Caused by Leishmania guyanensis: A Case-Control Study in the State of Amazonas, Brazil
Source: Pathogens. 2021 Apr 20;10(4):498. doi: 10.3390/pathogens10040498 (PMC8074324; doi:10.3390/pathogens10040498)
Supplement: Supplementary file 1 [file pathogens-10-00498-s001.zip › pathogens-1101723 supp fig S2.pdf]

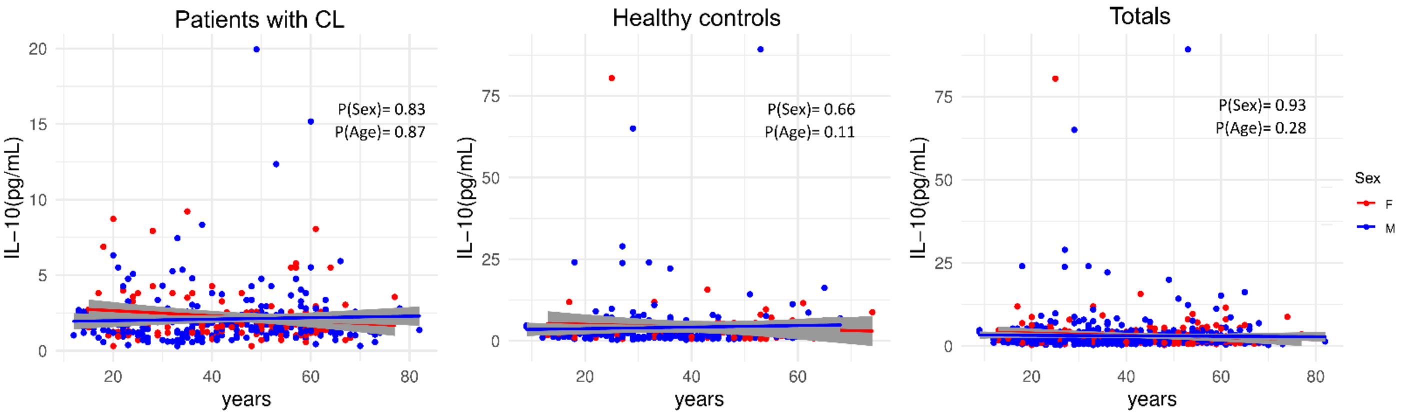

Supplementary Figure S2. Linear regression correlation analysis to show whether gender and age influence plasma IL-10 levels in patients with CL, healthy controls, and totals. The *p*-value for covariates Sex and Age (years) are shown in the figure. The colors of dots and lines (male in blue and female in red) represent the gender, and the 95% confidence interval is represented by gray color for each group. *p*-value <0.05 is considered statistically significant.
