# Supplementary material for: TREM1 rs2234237 (Thr25Ser) Polymorphism in Patients with Cutaneous Leishmaniasis Caused by Leishmania guyanensis: A Case-Control Study in the State of Amazonas, Brazil
Source: Pathogens. 2021 Apr 20;10(4):498. doi: 10.3390/pathogens10040498 (PMC8074324; doi:10.3390/pathogens10040498)
Supplement: Supplementary file 1 [file pathogens-10-00498-s001.zip › Supplementary Table 1 17032021.pdf]

Supplementary Table S1. Circulating plasma cytokines levels in patients with CL and healthy controls adjusted by sex and age.

Cases	IL-1β						IL-6						IL-8						IL-10						MCP-1						TNF-α					
**Codominant**	n	me	se	*p*-value	Corrected *p*-value	*q*-value	n	me	se	*p*-value	Corrected *P*-value	*q*-value	n	me	se	*p*-value	Corrected *P*-value	*q*-value	n	me	se	*p*-value	Corrected* *P*-value	*q*-value	n	me	se	*p*-value	Corrected *P*-value	*q*-value	n	me	se	*p*-value	Corrected *P*-value	*q*-value
**A/A**	234	1.1096	0.09829	0.41	0.52	0.88	238	0.6999	0.03162	0.92	0.92	0.93	245	1.805	0.07382	0.70	0.73	0.91	244	3.846	0.3493	0.29	0.42	0.88	184	8.486	0.6880	0.64	0.80	0.91	225	25.64	1.568	0.45	0.89	0.88
**A/T**	66	0.8494	0.05760				78	0.7265	0.06313				77	1.913	0.12592				71	2.940	0.2154				53	7.280	0.6264				65	25.62	2.927			
**T/T**	5	1.2660	0.31799				4	0.7425	0.08788				5	1.522	0.32018				5	5.012	1.1503				4	9.462	2.7344				5	38.50	6.806			
**Dominant**																																				
**A/A**	234	1.1096	0.09829	0.23	0.52	0.88	238	0.6999	0.03162	0.69	0.92	0.91	245	1.805	0.07382	0.60	0.73	0.90	244	3.846	0.3493	0.21	0.42	0.88	184	8.486	0.6880	0.40	0.80	0.88	225	25.64	1.568	0.89	0.89	0.93
**A/T-T/T**	71	0.8787	0.05859				82	0.7273	0.06015				82	1.890	0.11995				76	3.076	0.2205				57	7.433	0.6103				70	26.54	2.780			
**Recessive**																																				
**A/A-A/T**	300	1.0524	0.07791	0.80	0.80	0.92	316	0.7065	0.02841	0.92	0.92	0.93	312	1.831	0.06369	0.59	0.73	0.90	315	3.642	0.2756	0.53	0.53	0.89	237	8.216	0.5526	0.82	0.82	0.93	290	25.64	1.380	0.21	0.89	0.88
**T/T**	5	1.2660	0.31799				4	0.7425	0.08788				5	1.522	0.32018				5	5.012	1.1503				4	9.462	2.7344				5	38.50	6.806			
**Overdominant**																																				
**A/A-T/T**	239	1.1129	0.09642	0.18	0.52	0.88	242	0.7006	0.03112	0.70	0.92	0.92	250	1.800	0.07262	0.49	0.73	0.88	249	3.869	0.3431	0.14	0.42	0.88	188	8.507	0.6753	0.35	0.80	0.88	230	25.92	1.544	0.80	0.89	0.92
**A/T**	66	0.8494	0.05760				78	0.7265	0.06313				77	1.913	0.12592				71	2.940	0.2154				53	7.280	0.6264				65	25.62	2.927			
**Log-Additive**																																				
**0,1,2**				0.31	0.52	0.88				0.70	0.92	0.91				0.73	0.73	0.92				0.33	0.42	0.88				0.48	0.80	0.88				0.64	0.89	0.91
**Healthy Controls**																																				
**Codominant**																																				
**A/A**	251	0.6202	0.04669	0.66	0.77	0.91	255	0.4052	0.02091	0.80	0.91	0.92	255	1.156	0.04703	0.64	0.80	0.91	250	2.235	0.1339	0.36	0.45	0.88	219	4.821	0.2719	0.70	0.77	0.91	234	15.60	1.0959	0.72	0.82	0.92
**A/T**	97	0.7069	0.09455				102	0.4195	0.03499				101	1.238	0.08039				99	1.926	0.1179				88	5.153	0.3827				83	14.37	1.5700			
**T/T**	10	0.5730	0.08416				11	0.3509	0.04241				10	1.199	0.15362				11	2.061	0.3950				8	4.325	0.9907				9	14.09	2.4186			
**Dominant**																																				
**A/A**	251	0.6202	0.04669	0.45	0.77	0.88	255	0.4052	0.02091	0.91	0.91	0.93	255	1.156	0.04703	0.35	0.66	0.88	250	2.235	0.1339	0.16	0.34	0.88	219	4.821	0.2719	0.60	0.77	0.90	234	15.60	1.0959	0.42	0.75	0.88
**A/T-TT**	107	0.6244	0.08608				113	0.4128	0.03187				111	1.235	0.07430				110	1.939	0.1126				96	5.084	0.3599				92	14.34	1.4332			
**Recessive**																																				
**A/A-A/T**	348	0.6443	0.04274	0.77	0.77	0.92	357	0.4093	0.01795	0.54	0.91	0.89	365	1.179	0.04067	0.92	0.92	0.93	349	2.147	0.1017	0.87	0.87	0.93	307	4.916	0.2227	0.59	0.77	0.90	317	15.28	0.9067	0.82	0.82	0.93
**T/T**	10	0.5730	0.08416				11	0.3509	0.04241				10	1.199	0.15362				11	2.061	0.3950				8	4.325	0.9907				9	14.09	2.4186			
**Overdominant**																																				
**A/A-T/T**	261	0.6184	0.04501	0.37	0.77	0.88	266	0.4030	0.02012	0.73	0.91	0.92	265	1.158	0.04559	0.35	0.66	0.88	261	2.227	0.1292	0.16	0.34	0.88	227	4.803	0.2644	0.47	0.77	0.88	243	15.54	1.0588	0.45	0.75	0.88
**A/T**	97	0.7069	0.09455				102	0.4195	0.03499				101	1.238	0.08039				99	1.926	0.1179				88	5.153	0.3827				83	14.37	1.5700			
**Log-Additive**																																				
**0,1,2**				0.57	0.77	0.90				0.91	0.91	0.93				0.40	0.66	0.88				0.20	0.34	0.88				0.77	0.77	0.92				0.44	0.75	0.88
**Totals**																																				
**Codominant**																																				
**A/A**	487	0.8720	0.05679	0.59	0.74	0.90	503	0.5449	0.02000	0.69	0.75	0.91	504	1.474	0.04593	0.62	0.79	0.91	492	2.910	0.1412	0.05	0.10	0.88	389	6.849	0.3806	0.26	0.32	0.88	460	20.74	0.9995	0.24	0.41	0.88
**A/T**	167	0.7031	0.06636				179	0.5336	0.03018				181	1.536	0.07637				174	2.353	0.1219				139	5.856	0.3415				147	18.40	1.3959			
**T/T**	14	0.8443	0.14676				15	0.4553	0.05938				15	1.307	0.14674				15	3.141	0.5672				12	6.097	1.2525				13	24.04	4.3760			
**Dominant**																																				
**A/A**	487	0.8720	0.05679	0.31	0.61	0.88	503	0.5449	0.02000	0.57	0.75	0.90	504	1.474	0.04593	0.63	0.79	0.91	492	2.910	0.1412	0.03	0.09	0.88	389	6.849	0.3806	0.10	0.20	0.88	460	20.74	0.9995	0.21	0.41	0.88
**A/T-TT**	181	0.7878	0.06220				194	0.5276	0.02823				196	1.518	0.07148				189	2.415	0.1214				151	5.875	0.3286				160	18.86	1.3327			
**Recessive**																																				
**A/A-A/T**	654	0.8493	0.04556	0.98	0.98	0.94	682	0.5420	0.01673	0.44	0.75	0.88	685	1.491	0.03934	0.47	0.79	0.88	666	2.764	0.1094	0.59	0.70	0.90	528	6.587	0.2950	0.74	0.74	0.92	607	20.18	0.8300	0.43	0.43	0.88
**T/T**	14	0.8443	0.14676				15	0.4553	0.05938				15	1.307	0.14674				15	3.141	0.5672				12	6.097	1.2525				13	24.04	4.3770			
**Overdominant**																																				
**A/A-T/T**	501	0.8712	0.05535	0.30	0.61	0.88	518	0.5423	0.01950	0.75	0.75	0.92	519	1.469	0.04480	0.47	0.79	0.88	507	2.917	0.1380	0.01	0.06	0.88	401	6.826	0.3710	0.11	0.20	0.88	473	20.83	0.9792	0.12	0.41	0.88
**A/T**	167	0.7831	0.06636				179	0.5336	0.03018				181	1.536	0.07637				174	2.353	0.1219				139	5.856	0.3415				147	18.40	1.3959			
**Log-Additive**																																				
**0,1,2**				0.37	0.61	0.88				0.47	0.75	0.88				0.83	0.83	0.93				0.07	0.10	0.88				0.12	0.20	0.88				0.38	0.43	0.88
**Homozygous**																																				
**A/A**																			492	2.910	0.1412	0.76	0.76	0.92												
**T/T**																			15	3.141	0.5672															

me: mean (pg/mL); se: standard error; $p<0.05$ is considered significative., Corrected *p*-value: Benjamini and Hochberg's for strong control of the false discovery rate assuming five tests (codominant, dominant, recessive, overdominant, additive,); Corrected *p*-value*: Benjamini and Hochberg's for strong control of the false discovery rate assuming six tests (codominant, dominant, recessive, overdominant, additive, and homozygous) and FDR *q*-value.
